# Thirty-Day Outcomes From Standalone Minimally Invasive Surgery-Transforaminal Lumbar Interbody Fusion Patients in an Ambulatory Surgery Center vs. Hospital Setting

**DOI:** 10.7759/cureus.10197

**Published:** 2020-09-02

**Authors:** Scott Schlesinger, Kimberly Krugman, Diana Abbott, Jeffrey Arle

**Affiliations:** 1 Neurosurgery, Legacy Spine and Neurological Specialists and Legacy Surgery Center, Little Rock, USA; 2 Clinical Research, Wenzel Spine, Austin, USA; 3 Department of Biostatistics and Informatics, University of Colorado-Anschutz, Denver, USA; 4 Neurosurgery, Beth Israel Deaconess Medical Center, Harvard Medical School, Boston, USA

**Keywords:** standalone, outpatient, lumbar fusion, mis-tlif

## Abstract

Objectives

We sought to evaluate differences in perioperative baseline characteristics, operative efficiency, and 30-day safety events for patients undergoing standalone minimally invasive surgery-transforaminal lumbar interbody fusion (MIS-TLIF) in a hospital versus an ambulatory surgery center (ASC).

Methods

Patients were retrospectively identified and sequentially enrolled from the office records of a single, community neurosurgeon. Records for the first 50 qualifying patients in the hospital and ASC cohorts were retrieved. Variables collected included: baseline demographic and health status, operative safety (intra-op complications) and efficiency (operative time, fluoroscopy time, etc.), and 30-day post-operative safety (emergency room visits, re-admission, and re-operation).

Results

At baseline, hospital and ASC patients were equivalent in gender distribution, BMI, and pre-operative narcotic use. Statistically significant differences were found in age and comorbidity burden (ASA status and Charleson Comorbidity Index); p < 0.0001, p = 0.0039, and p < 0.001 respectively. The only significant difference in construct type between hospital and ASC patients was the proportion of one- versus two-level fusions; all two-level fusions were performed in the hospital group. There were no differences in operative time, need for transfusions, or iatrogenic complications. There were also no differences between the groups in 30-day events of ER visits, re-admission, re-operation, or post-operative narcotic refill use. The length of stay was significantly different between the ASC and hospital settings (p < 0.0001).

Conclusions

As expected, ASC patients were younger and relatively healthier compared to their hospital counterparts. Thirty-day safety events of ER visits, re-admission, re-operation, and narcotic refill utilization were representative of population norms. Patients with standalone, expandable MIS-TLIF underwent efficient operative procedures and experienced minimal 30-day complications independent of their operative status. ASC patients had the added benefit of significantly reduced length of stay over their hospital counterparts. Given the equivalency of the 30-day post-operative course for both patient cohorts, a substantial reduction in economic burden is likely for the ASC patients.

## Introduction

Over the past several years, a gradual shift has been made toward lumbar fusion approaches that are less invasive and theoretically protective of the thecal sac and nerve roots. Several authors have reported on their experiences of operative and perioperative (30-day) safety via a transforaminal lumbar interbody fusion (TLIF) approach [1-6], and more recently, studies examining the safety of outpatient lumbar fusion are on the rise [7-16]. Depending on the study, complications can be significantly under-reported. Often authors will report a handful of complications without clarifying whether their report is everything identified or only what they determined to be significant. The same is true regarding parameters of operative efficiency and the early post-operative course where surgical time, hospital stay, drain removal, and blood loss are intermittently reported at best. We sought to evaluate comprehensive potential differences in perioperative baseline characteristics, operative efficiency, and 30-day safety events for patients undergoing minimally invasive surgery-transforaminal lumbar interbody fusion (MIS-TLIF) in a hospital and an ambulatory surgery center (ASC) using an expandable, standalone titanium interbody device.

## Materials and methods

Patient selection

This was a retrospective study and approved through a central IRB (Pearl IRB, Indianapolis, USA). The senior author has been performing standalone MIS-TLIF procedures since 2011 and we chose to collect a convenience sample of the first 50 consecutive patients in each of the ASC and hospital settings. Patients were eligible for inclusion provided the expandable interbody was used on-label, they were over 18 years old at the time of surgery, did not have metal allergies, and there was no evidence of osteoporosis in their medical record (T-score of -2.5 or lower or Z-score of -2 or lower). 

Patient data were collected from July of 2011 to August of 2018. Relevant medical histories and comorbidities were gathered from office notes prior to surgery and all patient records (clinic visits, emails, phone calls, etc.) were collected for the first 30 days post-surgery. All operations were performed by a single surgeon with extensive experience of MIS-TLIF in both the hospital and ASC settings.

Operative technique

All MIS-TLIF procedures were performed utilizing the same surgical protocol at the ASC and hospital. With the patient in the prone position on a radiolucent table and using standard dual planar fluoroscopy, the side for decompression was selected based on the patient’s primary symptoms. The appropriate level was identified utilizing a spinal needle and fluoroscopic imaging. A roughly 3-cm incision was made approximately 4 to 5 cm off midline in a paramedian fashion. Once the facial layer was incised, a tubular retractor system was used and sequential dilation of the paraspinous soft tissue was completed in a Wiltse fashion. Using the microscopic technique, neural decompression was performed by bilateral or unilateral laminectomy as indicated, followed by a complete annulotomy and discectomy as well as a complete foraminal decompression as far lateral as necessary to completely decompress the exiting nerve root.

**Figure 1:**
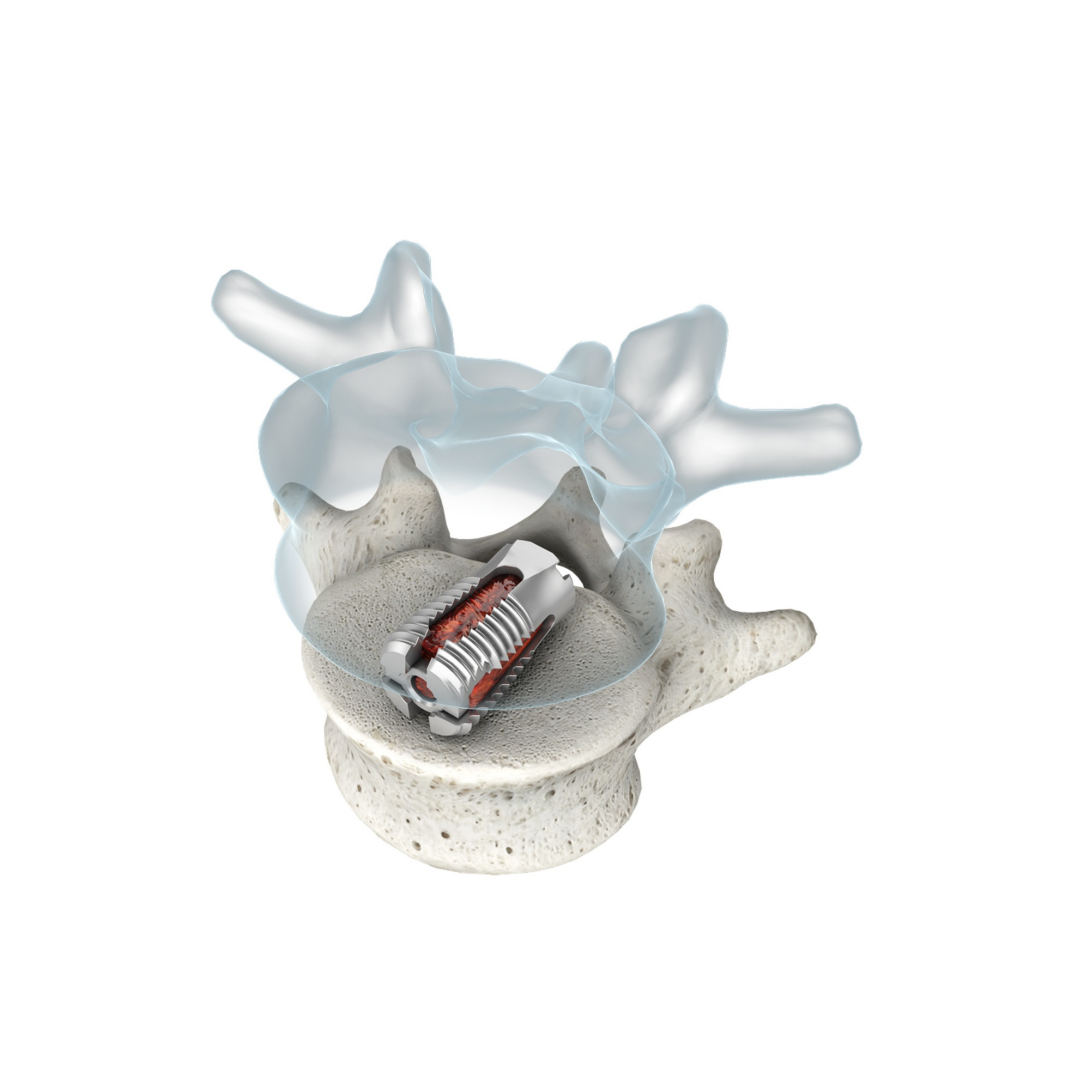
Illustration of the VariLift-LX device in a TLIF application. Rendering provided by Wenzel Spine, Inc. TLIF: transforaminal lumbar interbody fusion.

An expandable distraction device was inserted into the disk space to determine the optimal size for the interbody implant. The appropriately sized interbody distractor and obturator were then inserted in a neutral orientation and rotated 90 degrees to distract the vertebral bodies to the previously determined spacing. The obturator was removed, and the nerve root protector was placed to retract the exiting nerve root superiorly. The threaded trial/tap was advanced in conjunction with fluoroscopic imaging to confirm the proper sizing of the interbody device and to prepare the vertebral body endplates for the permanent device. The appropriately sized interbody device (VariLift-LX Wenzel Spine, Austin, USA) was then threaded into position with fluoroscopic guidance and expanded. The use of this expandable interbody has been described elsewhere and represents the only expandable interbody approved for standalone use by the US FDA [17-22]. After placement and expansion, the device was filled with morselized autograft from the harvested lamina mixed with allograft bone product or DBM if needed. A complete foraminotomy was performed at this level to ensure that the exiting nerve root was free after distraction from medial to the far lateral aspect of the neural foramen. A posterior lateral fusion was carried out with decortication of the contralateral lamina if the laminectomy was not bilateral in nature and/or with decortication of the ipsilateral transverse processes or sacral ala plus or minus contra-laterally as well. The surgical site was irrigated and closed in the standard technique over a drain placed in the epidural space. Figure 1 shows a rendering of this interbody within a TLIF construct.

Post-op protocol

Patients in the ASC setting were discharged within four hours and most in the hospital were discharged the following day. Drains were left until the output after 20 hours was ≤ 5 cc/hour. Patients and their families were instructed in the care and reporting of drain output. Patients were instructed on lifting precautions, brace usage, and movement precautions (flexion/extension) for the first six months post-op. Patients were asked to ambulate often; at least once every hour while awake. Clinic follow-up intervals: drain removal (approximately one to five days post-op), six weeks, three months, and six months. The collection of routine X-rays starts at the six-week visit.

Statistical Analysis

A variety of categorical and continuous variables were collected. Summary measures for continuous variables have been presented as means ± standard deviation and categorical data as proportions. The primary interest was to compare baseline, operative, and post-operative characteristics between the study groups and hypothesis testing focused on revealing differences or similarities between these cohorts. For categorical measures a hypothesis test for a difference in proportion between the two settings was conducted using a chi-square test and p-values are reported. When cell counts were small, the Fisher’s exact test p-value is reported. To assess differences in continuous variables between the two settings, an analysis of variance (AOV) model was run to compare mean values, and p-values from the comparison are reported. For all variables except estimated blood loss, model assumptions of normality were met. To compare estimated blood loss across the two settings, a log transformation was first performed prior to running the AOV model.

## Results

The first 50 eligible sequential expandable interbody patients were entered into data collection for the ASC cohort over the period from July of 2011 to February of 2018. Similarly, 50 eligible sequential hospital patients were entered into data collection from October of 2011 to August of 2018. The operative window is large as the lead surgeon began using this implant in 2011 and early 2012 and then resumed using the implant in 2017. Indications for surgery included neural foraminal stenosis, canal stenosis, lateral recess stenosis, spondylolisthesis, and recurrent herniated disk. The comparison of patient baseline characteristics can be found in Table 1. ASC patients were statistically younger, had a lower comorbidity burden, and higher functional status compared to their hospital counterparts. No difference was found in pre-operative narcotic use between the groups and records did not allow for conversion of milligram morphine equivalents (MME) prior to surgery.

**Table 1:** Baseline characteristics of ASC and hospital patients. CCI: Charlson Comorbidity Index, ASA: American Society of Anesthesiologists Physical Status Classification System.

Attribute		ASC	Hospital	Differences
Gender				p = 0.687
Female		27 (54%)	29 (58%)	
Male		23 (46%)	21 (42%)	
Age				p < 0.0001
Mean ± SD		51.02 ± 9.19	64.2 ± 13.26	
Race				p = 0.4380
Caucasian		42 (84%)	41 (82%)	
African American		3 (6%)	1 (2%)	
Asian American		0 (0%)	0 (0%)	
Native American		0 (0%)	0 (0%)	
Native Hawaiian or Pacific Islander		0 (0%)	0 (0%)	
Unknown		5 (10%)	8 (16%)	
BMI				p = 0.7528
Mean ± SD		31.88 ± 7.34	32.33 ± 6.83	
Smoking status				p = 0.6348
Yes		16 (32%)	14 (28%)	
No		33 (66%)	33 (66%)	
Unknown		1 (2%)	3 (6%)	
ASA				p = 0.0039
1		2 (4%)	0 (0%)	
2		35 (70%)	25 (50%)	
3		11 (22%)	25 (50%)	
4		0 (0%)	0 (0%)	
Missing data		2 (4%)	0 (0%)	
CCI				p ≤ 0.0001
0		19 (38%)	7 (14%)	
1		13 (26%)	4 (8%)	
2		15 (30%)	11 (22%)	
3		1 (2%)	16 (32%)	
4		1 (2%)	10 (20%)	
5		1 (2%)	1 (2%)	
6		0 (0%)	1 (2%)	
Functional dtatus				p = 0.0039
Ambulatory		47 (94%)	41 (82%)	
Cane assisted		0 (0%)	3 (6%)	
Walker assisted		0 (0%)	2 (4%)	
Wheelchair		0 (0%)	3 (6%)	
Unknown		3 (6%)	1 (2%)	
Opioid use				
Class II		16 (32%)	24 (48%)	p = 0.1025
Class III		0 (0%)	1 (2%)	p = 1.0000
Class IV		10 (20%)	12 (24%)	p = 0.6292
Overall		23 (46%)	30 (60%)	p = 0.1608

This study was not limited to one-level patients or standalone interbody constructs; Table 2 enumerates the type of fusion construct and proportions of one- versus two-level fusion patients. All two-level procedures were performed in the hospital setting. Only 5% of all patients had supplemental posterior fixation and, in each case, the posterior fixation was via pedicle screws and rods. Eight patients had a fusion that was adjacent to a prior fused level and the proportion of this occurrence was more common in the hospital patients than the ASC but did not reach statistical significance. In no case did a patient undergoing an adjacent segment fusion also have a supplemental posterior fixation. Four of the five patients receiving posterior instrumentation were in the early operative experience of the standalone interbody (operative years of 2011 and 2012); only one supplemental posterior fixation case happened in the more recent past (2017).

**Table 2:** Details of the types of spine constructs represented in each patient cohort. ASC: ambulatory surgery center, ASD: adjacent segment disease

Construct Details		ASC	Hospital	Differences
Operative level				
L2/3		0 (0%)	2 (4%)	p = 0.4949
L3/4		3 (6%)	9 (18%)	p = 0.0648
L4/5		18 (36%)	29 (58%)	p = 0.0275
L5/S1		29 (58%)	24 (48%)	p = 0.3164
Previous surgical intervention at the same level as current surgical intervention				
L3/4		1 (33%)	3 (33%)	
L4/5		3 (16.7%)	6 (20.6%)	
L5/S1		11 (37.9%)	5 (20.8%)	
One-level		50 (100%)	36 (72%)	p < 0.0001
Two-level		0 (0%)	14 (28%)	p < 0.0001
Supplemental Fixation (pedicle screws/rods)		3 (6%)	2 (4%)	p = 1.000
ASD		1 (2%)	7 (14%)	p = 0.0594

No differences were found between the cohorts in terms of operative time, the need for transfusion, or intraoperative complications. Estimated blood loss was found to be statistically higher in the ASC group yet the clinical relevance of this difference is not appreciable. Analysis of blood loss using only the more recent experience (2017 on) showed no significant differences between the groups (p = 0.1110). All intraoperative complications were incidental durotomies and one patient was assigned the status of ‘unknown’ for receiving any blood products due to incomplete records. Fluoroscopy records were not readily available for the ASC patients and the hospital fluoroscopy times are listed for the sake of completeness in Table 3. The single most significant difference between the ASC and hospital patients was their length of stay. On average, ASC patients were in the facility for one third of a day (eight hours) while the hospital patients were admitted for two days (p < 0.0001).

**Table 3:** Operative details from each patient cohort. *Data are log-transformed after performing an outlier analysis. ASC: ambulatory surgery center, ASD: adjacent segment disease

Operative Details		ASC	Hospital	Differences
Operative time (minute)				
Overall		126.7 ± 29.1	138.9 ± 38.9	p = 0.0808
One-level		126.7 ± 29.1	131 ± 39.5	p = 0.5678
Two-level		--	159.4 ± 29.8	n/a
Supplemental fixation		144.7 ± 39.4	187.5 ± 47.4	p = 0.3474
ASD		93	147.1 ± 58	n/a
Fluoroscopy time (seconds)				
Overall		--	34.3 ± 24.8	n/a
One-level		--	34.7 ± 28.2	n/a
Two-level		--	33.4 ± 14.6	n/a
Supplemental fixation		--	75 ± 38.2	n/a
ASD		--	49 ± 53.5	n/a
Estimated blood loss (cc’s)				
Overall		296 ± 235	187 ± 212	p < 0.0010*
One-level		296 ± 235	166 ± 230	p < 0.0010*
Two-level		--	238 ± 155	n/a
Supplemental fixation		350 ± 304	150	n/a
ASD		--	--	n/a
Transfusions				p = 1.000
Yes		0 (0%)	0 (0%)	
No		49 (98%)	50 (100%)	
Unknown		1 (2%)	--	
Operative complications				p = 0.1371
Yes		4 (8%)	9 (18%)	
No		46 (92%)	41 (82%)	
Length of stay (days)		0.29 ± 0.06	1.95 ± 1.27	p < 0.0001

Post-operatively, events in the first 30 days were captured and reported in Table 4. Patient touch points of clinic visits and phone calls were quantified. The mean number of clinic visits for the ASC patients was statistically higher compared to hospital patients (p = 0.0134); driven by the need for ASC patients to return to clinic for drain removal. Eighty-seven patients did not require an emergency room visit. In the case of six patients, limited records were identified as ‘unknown’ for the incidence of ER visits to be conservative in how these patients were categorized. Seven patients presented to the emergency room during the perioperative period with no statistical difference between the ASC and hospital cohorts. Causes for seeking emergency care included: two for pain with unremarkable findings on labs or imaging, one for a fall with pain to the leg and knee and unremarkable findings on imaging, one patient presented to two separate ERs claiming a fall and seeking pain medication, one remote patient went to the ER to have their drain removed, one patient with pre-existing stage IV renal disease became toxic and presented to the ER for transfusion, and one remote patient was referred to the ER by their primary care as being hypotensive with no evidence of hypotension at the ER. 

**Table 4:** Thirty-day follow-up events and their frequency in each cohort. ^†^Patients with limited records in the 30-day follow-up period were identified as ‘unknown’ in lieu of ‘no’ for ER visits. ASC: ambulatory surgery center.

Follow-Up Events		ASC	Hospital	Differences
Mean # of calls		2.08 ± 1.50	2.40 ± 1.60	p = 0.3047
Mean # of clinic visits		0.52 ± 0.54	0.26 ± 0.49	p = 0.0134
ER visits				p = 0.4464
No		43 (86%)	44 (88%)	
Yes		5 (10%)	2 (4%)	
Unknown^†^		2 (4%)	4 (8%)	
Re-admission				p = 0.2499
No		49 (98%)	47 (94%)	
Yes		1 (2%)	3 (6%)	
Re-operation				p = 1.000
No		49 (98%)	49 (98%)	
Yes		1 (2%)	1 (2%)	

Only four subjects were re-admitted during the first 30 days: one in the ASC cohort and three in the hospital cohort. The single patient re-admitted in the ASC cohort developed progressive numbness and loss of motor function in their legs over a weekend and did not call the on-call doctor. After being asked repeatedly to present to the ER, the patient presented two days later and was taken to surgery; this patient is represented as the single case of re-operation in the ASC cohort. Their wound was re-explored and a small seroma and hematoma evacuated. The patient’s numbness improved immediately. In the hospital cohort, three patients were re-admitted in the first 30 days. One of these patients was re-admitted for pain and an image-guided seroma evacuation was performed. The second patient was discharged from the hospital into a rehab facility in her town of residence. This patient was transferred back to the surgery hospital for wound healing issues, her wound was re-explored, and the implant found to be in place but loose in the space. The implant was removed and allograft bone packed into the disk space. The third patient requested she be admitted to a rehab facility as she could not care for herself during recovery and her husband was not able to care for her. There were no differences between ASC and hospital patients regarding the need for re-admission or re-operation.

Finally, an investigation into narcotic refills was undertaken between the groups. Eight ASC and three hospital patients were prescribed narcotic refills during the 30-day follow-up period with no difference in proportions between the groups. Records were complete to calculate MME (Table 5). There was no difference between the groups for MMEs during follow-up.

**Table 5:** Thirty-day narcotic refills prescribed as well as the MME units of the refills for each cohort. ASC: ambulatory surgery center, MME: milligram morphine equivalent.

Narcotic Refills	ASC	Hospital	Differences
Refills (n, %)	8 (16%)	3 (6%)	p = 0.1100
MME	55.5 ± 17.8	54 ± 0	p = 0.8897

-

## Discussion

Direct reports of the surgeon and patient experience with outpatient lumbar fusion procedures have been scarce and no prospective studies of this topic have been identified. In 2014, two different author groups reported their experiences with outpatient lumbar fusion [8,9]. Chin et al. reviewed medical records from 16 patients who had undergone single-level posterior lumbar interbody fusion (PLIF) or TLIF with posterior supplemental fixation operated at an ASC [8]. Eckman et al. reviewed 728 patients who were eligible and elected to go home on the same day as fusion and compared them to a group of 277 patients who were required to stay at least overnight due to their age (65 years and over) or due to comorbid medical conditions [9]. Patients in the study of Chin et al. were much younger than our ASC cohort (mean of 42.8 years vs. 51 years), yet other baseline and operative parameters were on par with our data (similar surgical times, blood loss, and BMI). Chin et al. followed their patients longer prior to reporting and were able to share a pseudarthrosis rate of 12.5% for two of their patients who were heavy smokers [8]. Ultimately, revision surgery was needed for these patients. They also reported a single post-operative complication of infection (6.3%) treated by oral antibiotics. They had no hospital admissions for pain control post-op, yet these patients were treated at a time where using Oxycontin and Percocet did not carry the significance these medications command today. In general, the authors concluded that carefully selected patients could be safely offered outpatient spine fusion.

The report by Eckman et al. evaluated a much larger cohort of patients who had undergone one- or two-level MIS-TLIF with unilateral posterior fixation [9]. These patients had surgery in a hospital and were given the choice to go home the same day of surgery provided they were under 65 years old and elected to be discharged. In this study, the patients choosing to leave the day of surgery were similar in age to our ASC cohort (52 years old vs. 51 years). The hospital patients were approximately the same age as our hospital cohort (64 years old vs. 64.2 years). The authors reported on patient events for the first three post-operative months, yet only major medical complications and re-admissions for the first two weeks post-op. Eckman et al. found the hospital patients to have significantly more transfusions than the outpatient group, and while not statistically significant, the hospital group had almost twice as many re-operations on average compared to the outpatients (2.2% vs. 4.7%). These authors further stratified outpatient and hospital groups by age (those less than 65 years in each cohort and then those over 65 years in each cohort) and found no differences in the proportion of early medical complications and re-admissions between hospital and outpatient for the ‘younger’ patients. Conversely, the ‘older’ hospital patients had significantly more early complications and re-admissions compared to the outpatient group despite the fact that the ‘older’ outpatient group has a higher comorbidity burden compared to the hospital group. 

In 2016, Emami et al. reported on their experience with MIS-TLIF outpatient surgery [10]. These authors retrospectively reviewed 32 patients who were discharged in less than 24 hours compared to 64 patients who were admitted and considered as inpatients. Similar to our experience, these authors found the outpatient group to be younger and healthier (both ASA and CCI). The length of stay was significantly different between the groups with outpatients staying on average 0.6 days versus their inpatient counterparts staying for 2.6 days. The authors reported on post-operative complications, yet the interpretation of re-admissions and the need for surgical revision is difficult to ascertain. They found no differences in the proportion of re-admissions between the groups (3% for outpatient and 4.7% for inpatient). The three re-admissions in the outpatient group were all re-operations arising from graft or hardware issues. For the inpatient group, understanding re-admissions that are directly linked to a re-operation are difficult as the authors report in generic terms that re-operations happened for some patients while they were still under the index surgery admission and then go on to report the details of two re-operations that happened in the hospital cohort after the initial discharge. Ultimately, the authors concluded that comparable clinical and safety outcomes were found between the groups, and therefore MIS-TLIF may be safely performed as an outpatient procedure.

In 2016, Chin et al. again reported on outpatient lumbar fusions and compared a patient series to a hospital counterpart [11]. In this report, the comparison was less about possible differences between inpatient and outpatient groups and more about the anatomic trajectory of posterior pedicle screws. Here, they retrospectively reviewed 30 patients who underwent surgery center fusions with a cortical bone trajectory compared to 30 hospital patients with traditional pedicle screw placement. In this publication, patients were followed for two years and fusion assessments were made between the groups. The authors reported no major complications and no unplanned post-operative admissions. Their mean operative time for the surgery center patients compared closely to our experience of outpatient surgery (mean of 138 minutes vs. 127 minutes). Overall, the authors concluded a demonstration of successful conversion from hospital to surgery center lumbar fusions based on their less exposure technique due to the implementation of cortical bone trajectory pedicle screws.

While prior reports have provided a glimpse into the outpatient experience with lumbar interbody fusion, there remain disparities between the publications: MIS versus open approaches, pedicle screw trajectory, and discharge from a surgery center versus an under 24-hour discharge from a hospital. Other researchers have approached the topic from a population-based view and leveraged national databases to examine the safety and efficacy of outpatient lumbar fusion. Bovonratwet et al. interrogated the ACS-NSQIP database for the years 2005-2015 to evaluate possible differences in outpatient posterior fusions versus inpatient posterior fusions by defining inpatient as a length of stay greater than 0 days [12]. Their query returned an outpatient sample of 360 cases with statistical differences in age, gender, and ASA status pre-operatively compared to inpatient. They then employed propensity score matching to evaluate inpatient versus outpatient differences for matched groups and found all statistically significant differences vanished. The same was true for 30-day perioperative events except for blood transfusion which remained statistically higher in the inpatient group. They report unadjusted proportions of 3.6% versus 5.4% re-admission and 1.1% versus 2.3% return to the OR (outpatient vs. inpatient).

In a similar study of the ACS-NSQIP, Garcia et al. reviewed single-level TLIF surgery from 2011 to 2013 with the intent of evaluating 30-day re-admission risk factors [13]. This study did not stratify by patient discharge status. Of the 4992 patients who met inclusion criteria, 275 (5.5%) were re-admitted within 30 days of their index TLIF procedure and 179 (3.6%) required re-operation of their index TLIF procedure. Following the work by Garcia et al. [13], Katz et al. reported their review of the ACS-NSQIP database comparing anterior to posterior fusion approaches and the implications to 30-day outcomes [14]. PLIF and TLIF patients were grouped together into posterior fusion and ALIF with LLIF patients were grouped into anterior fusion. All patients had interbody fusion and a maximum of two instrumented levels. Their review covered the years 2005-2015. Revision surgery was 5.9% for the posterior fusion group versus 4.4% for the anterior fusion group. Re-admission was found to be 5% for both groups and re-operation was 3.1% for posterior fusion and 3.2% for anterior fusion.

Arshi et al. used the Humana claims database to review trends and complications of one- or two-level posterior lumbar fusion for the outpatient and inpatient experience [15]. These authors took a longer view to look into post-operative complications. In this study, PLIF and TLIF were grouped together and records for the years 2007-2015 were reviewed. These authors reviewed complications up to one year post-operatively. They found that at one-year, outpatient PLF was associated with a higher risk of posterior revision or extension of fusion, conversion to anterior fusion, and stenosis requiring decompressive laminectomy. These authors proposed that the increased risks for outpatients may come from the minimally invasive approach itself; limited visualization and possibly incomplete decompression leading to future pain and symptoms. 

We report these various studies, both direct research and database reviews, to benchmark the present experience of a single, community neurosurgeon who is performing the same MIS-TLIF procedure both at a hospital and in an ASC environment. Our re-admission proportion of 2% for the ASC setting and 6% for the hospital patients is well aligned both with direct retrospective series as well as on a population-based view of these procedures. The same is true for re-operation proportions found in our ASC and hospital cohorts of 2% each. Our data are further stratified and reported compared to previous accounts and we found that seven patients (five ASCs and two hospitals) presented to an emergency room in the first 30 days post-op with only two of these incidents being directly related to their surgery (continued pain), yet with unremarkable findings on labs or imaging. The only difference noted between our ASC and hospital patients during the follow-up period was the number of clinic visits associated with each segment. All patients received a drain, and while the hospital patients were often able to have the drain removed before their discharge, the ASC patients all had to return for drain removal. 

The drive for both MIS procedures and outpatient surgery comes from a desire to optimize the surgical experience for all stakeholders. Less invasive surgeries are often better for the patient from a complication standpoint and cheaper for the healthcare system by reducing the incidence of events such as infection and reducing the length of stay. Moving surgery to an outpatient setting is another form of providing a minimally invasive experience to the patient. While our objective was not to review a link between outpatient fusion and possible cost savings, we found one recent report to be particularly relevant to our experience.

In 2019, Hartman et al. retrospectively reviewed 20 patients who had undergone standalone lateral interbody fusion (LIF) or MIS-TLIF [16]. The authors were interested in cost differentials between these two approaches. Ten patients underwent MIS-TLIF with unspecified unilateral or bilateral posterior fixation and ten patients underwent LIF (lateral lumbar interbody fusion) as a standalone procedure. In this study, there were no re-admissions or return to the operating room for any patient. The authors reported their OR time to be 286 minutes for TLIF and 131 minutes for LIF. Operative times of our MIS-TLIF patients are roughly equivalent to the reported experience for their LIF patients (127 vs. 131 minutes, respectively). All the subjects in our study were instrumented with a standalone expandable titanium interbody without integrated screws and which does not require the use of supplemental fixation. When Hartman et al. evaluated costs, they found statistically significant reductions for their LIF group compared to the TLIF group for total, variable, and fixed costs. It is conceivable that the patients in our ASC cohort could have similar costs (and cost savings) to their LIF patients [16].

The present study has limitations including the retrospective nature and derivation from a single surgeon and single geography. The reporting of only perioperative outcomes without long-term follow-up is also a limitation to this work; at the time of writing, many of the patients represented by this study have not yet reached their two-year follow-up for traditional fusion assessment. Our objective was to add to the evidence available in reporting MIS-TLIF perioperative results and the experience of patients in both a hospital and ASC setting. Future research will investigate the long-term outcomes from these standalone fusion patients (fusion rates at 12 and 24 months, patient disposition at 12 and 24 months, and long-term revision/re-operation rates) as well as economic implications of both the instrumentation and the point of care (outpatient vs. inpatient).

## Conclusions

This represents the first TLIF publication of a standalone expandable interbody. We have found, like others before, performing lumbar fusion on appropriate patients in an ASC facility is safe and effective in the perioperative period. The standalone MIS-TLIF procedure can be performed without the need to modify a fusion technique (i.e., through screw trajectory) and when performed in an ASC allows for a significant reduction in the length of stay. Our outpatients had a re-admission proportion of 2% and a re-operation proportion of 2%; well below the upper bounds previously published. Standalone MIS-TLIF can be performed on-label, as an outpatient surgery, and with a significant reduction in the total implanted hardware (likely to compound cost savings).

## References

[1] de Kunder SL, van Kuijk SM, Rijkers K, Caelers IJ, van Hemert WL, de Bie RA, van Santbrink H (2017). Transforaminal lumbar interbody fusion (TLIF) versus posterior lumbar interbody fusion (PLIF) in lumbar spondylolisthesis: a systematic review and meta-analysis. Spine J.

[2] Liu J, Deng H, Long X, Chen X, Xu R, Liu Z (2016). A comparative study of perioperative complications between transforaminal versus posterior lumbar interbody fusion in degenerative lumbar spondylolisthesis. Eur Spine J.

[3] Lee HJ, Kim JS, Ryu KS (2016). Minimally invasive TLIF using unilateral approach and single cage at single level in patients over 65. Biomed Res Int.

[4] Lan T, Hu SY, Zhang YT, Zheng YC, Zhang R, Shen Z, Yang XJ (2018). Comparison between posterior lumbar interbody fusion and transforaminal lumbar interbody for the treatment of lumbar degenerative diseases: a systematic review and meta-analysis. World Neurosurg.

[5] Levin JM, Tanenbaum JE, Steinmetz MP, Mroz TE, Overley SC (2018). Posterolateral fusion (PLF) versus transforaminal lumbar interbody fusion (TLIF) for spondylolisthesis: a systematic review and meta-analysis. Spine J.

[6] Wong AP, Smith ZA, Nixon AT (2015). Intraoperative and perioperative complications in minimally invasive transforaminal lumbar interbody fusion: a review of 513 patients. J Neurosurg Spine.

[7] Basques BA, Ferguson J, Kunze KN, Phillips FM (2019). Lumbar spinal fusion in the outpatient setting: an update on management, surgical approaches and planning. J Spine Surg.

[8] Chin KR, Coombs AV, Seale JA (2015). Feasibility and patient-reported outcomes after outpatient single-level instrumented posterior lumbar interbody fusion in a surgery center. Spine.

[9] Eckman WW, Hester L, McMillen M (2014). Same-day discharge after minimally invasive transforaminal lumbar interbody fusion: a series of 808 cases. Clin Orthop Relat Res.

[10] Emami A, Faloon M, Issa K, Shafa E, Pourtaheri S, Sinha K, Hwang KS (2016). Minimally invasive transforaminal lumbar interbody fusion in the outpatient setting. Orthopedics.

[11] Chin KR, Pencle FJ, Coombs AV (2016). Lateral lumbar interbody fusion in ambulatory surgery centers: patient selection and outcome measures compared with an inhospital cohort spine. Spine.

[12] Bovonratwet P, Ottesen TD, Gala RJ, Rubio DR, Ondeck NT, Mclynn RP, Grauter MD (2018). Outpatient elective posterior lumbar fusions appear to be safely considered for appropriately selected patients. Spine J.

[13] Garcia RM, Khanna R, Dahdaleh NS, Cybulski G, Lam S, Smith Z (2017). Thirty-day readmission risk factors following single-level transforaminal lumbar interbody fusion (TLIF) for 4992 patients from the ACS-NSQIP database. Global Spine J.

[14] Katz AD, Mancini N, Karukonda T, Greenwood M, Cote M, Moss I (2019). Approach-based comparative and predictor analysis of 30-day readmission, reoperation, and morbidity in patients undergoing lumbar interbody fusion using the ACS-NSQIP dataset. Spine.

[15] Arshi A, Park HY, Blumstein GW (2018). Outpatient posterior lumbar fusion: a population-based analysis of trends and complication rates. Spine.

[16] Hartman C, Hemphill C, Godzik J (2019). Analysis of cost and 30-day outcomes in single-level transforaminal lumbar interbody fusion and less invasive, stand-alone lateral transpsoas interbody fusion. World Neurosurg.

[17] Attia D (2020). PLIF using varilift expandable cages: more than 5 years experience of stand alone cages. Orthop Proc.

[18] Doria C, Lisai P, Meloni GB, Pala PP, Serra M, Fabbriciani C (2004). Instrumented posterior interbody fusion in degenerative and multioperated lumbar spine. J Orthopaed Traumatol.

[19] Neely WF, Fichtel F, del Monaco DC, Block JE (2016). Treatment of symptomatic lumbar disc degeneration with the VariLift-L interbody fusion system: retrospective review of 470 cases. Int J Spine Surg.

[20] Wohlfeld BJ, del Monaco DC (2017). Patient outcomes following posterior lumbar interbody fusion for adjacent segment disease using VariLift® as a standalone expandable interbody device. J Spine.

[21] Barrett-Tuck R, del Monaco DC, Block JE (2017). One and two level posterior lumbar interbody fusion (PLIF) using an expandable, stand-alone, interbody fusion device: a VariLift® case series. J Spine Surg.

[22] Lewandrowski K-U (2018). Surgical technique of endoscopic transforaminal decompression and fusion with a threaded expandable interbody fusion cage and a report of 24 cases. J Spine.

